# Clinical Practice Guidelines and Industry

**DOI:** 10.4103/0973-1229.32149

**Published:** 2007

In this section we shall see what Clinical Practice Guidelines (CPGs) should be and what they are, the recent case of Xigris and a thriller-like manipulation by the concerned company to enter a performance ‘bundle’, CPG effectiveness/cost effectiveness and other considerations, how they serve Industry needs, and what methods can possibly assist them actualise their enormous potential.

## Introduction

From the early nineties, a number of Clinical Practice Guidelines (CPGs) have been created and published by many different associations and organizations at considerable expense. CPGs are supposed to serve an important purpose. They offer objective consensus of expert opinion on treatment and hence are trusted by hospitals and practicing physicians alike. They can reduce the possibility of inappropriate care being delivered, while helping introduce new knowledge into clinical practice (Grimshaw and Russell, 1993; Merritt *et al.*, 1997; Woolf *et al.*, 1999). They are a distillate of biomedical wisdom at a certain point of time applied to better and more efficient patient care. Hence, rigorously developed guidelines can translate complicated research findings into actionable recommendations of clinical care (Shiffman *et al.*, 2003; Haines and Jones, 1994). Clinical practice guidelines have generally been accepted as an objective consensus on evidence (Baird, 2003). Practice guidelines approved by expert panels are intended to standardize care in such a way as to improve health outcomes (Eichacker *et al.*, 2006). Major hospitals and knowledge updated physicians feel reassured they are doing the very best by following CPGs. State of the art, and such other fancy labels, can be justifiably applied to them.

McMaster *et al.,* (2007) have talked recently of getting guidelines to work in practice. In an effort to make CPGs more effective, developers of such guidelines have started grouping evidence-based interventions into what are called ‘bundles’. The justification for this is that it helps condense multiple recommendations into a single protocol which can have a measurable effect on patient outcomes. Moreover, such bundled performance measures are readymade standards for insurance and other pay-for-performance initiatives, since these agencies can base reimbursement on compliance with such bundle components (Eichacker *et al.*, 2006).

All is fine up till here. Now the sordid part begins. Such ‘bundles’ become attractive recipes to rake in the moolah. What happens in practice is that pharma and medical device companies find these bundles a potentially powerful vehicle for promoting their products. So they have begun to invest in trying to influence the adoption of guidelines to serve their own goals (ibid).

Hence, these much vaunted and hallowed Guidelines become amenable to manipulation. We know industry tries to influence clinical trials. They also try influencing clinical practice guidelines which give recommendations on drugs, dosages and criteria for drug treatment and are intended to affect the practice of large number of physicians (Baird, 2003).

## CPG Bundles, Xigris, Eli Lilly And Marketing Strategies

A recent well-documented example of the same is the case of Eli Lilly and its recently launched Xigris, (recombinant human activated protein C or rhAPC, also known as drotrecogin alfa [activated]) and the development of guidelines for the treatment of sepsis, which was orchestrated as an extension of a pharmaceutical marketing campaign (Eichacker *et al.*, 2006). This is a case study straight out of a mystery thriller.

In 2001, US FDA approved Xigris for the treatment of sepsis. In 2002, Lilly hired Belsito and Company, a public relations firm, to formulate and launch a three-pronged marketing strategy to improve sales (ibid). (It would make a fit case study for marketing and management students about how strategies can be planned by them to boost sales at other places, but how they should *not* allow marketing and sales considerations to over-ride patient welfare in biomedicine, because it ultimately does not help the client's cause.)

## The Three-Pronged Strategy

What was this three-pronged strategy?

1. Target the physicians and medical trade media by various marketing initiatives.
2. Since it involved such a grave life and death condition as sepsis and since rhAPC was expensive, spread the word that the drug was being rationed and physicians were being ‘systematically forced’ to decide who would live and who die (Eichacker *et al.,* 2006). As part of this campaign, Lilly provided a $1.8 million grant to physicians and bio-ethicists to form a VERICC Task Force (VERICC stands for Values, Ethics and Rationing in Critical Care) meant to address ethical issues raised by rationing in the intensive care unit (ibid).
3. A Surviving Sepsis Campaign was established, in theory to raise awareness of severe sepsis and generate momentum towards the development of treatment guidelines (ibid). This had two parts: first part launched in the Oct 2002 meeting of the European Society of Intensive Care Medicine (ESICM); second part, launched in June 2003, where international experts in critical care and infectious diseases were convened to create guidelines for sepsis management, which were then published in *Critical Care Medicine* in March 2004. For both these initiatives, Lilly provided more than 90% of the funding and many participants had financial or other relationships with the company (ibid).

What Belsito essentially did was assemble the VERICC Task Force, help launch the Surviving Sepsis Campaign and initiate the media-outreach programme to ‘raise awareness’ of alleged rationing in severe sepsis with the intent of generating demand for rhAPC (ibid).

In other words, first a sensitization of clients by media outreach programmes, then generating a strong felt need by talking against rationing in critical care (and cleverly involving even bio-ethicists in this manoeuvre), then assembling opinion makers from the critical care field to mouth suitable phrases in favour of the product and thus generating a strong demand for a new product. All suitably sponsored. A clever, well orchestrated marketing strategy.

### What's Wrong With This Hype?

What was wrong with this hype? Why should we be concerned with it? The reasons are as follows:

1. The campaign for promotion of the drug was fine, but what was not was the fact that it failed to discuss persisting concerns about rhAPC use, which were reinforced by recent trials (ibid). For example, the Recombinant Protein C Worldwide Evaluation in Severe Sepsis (PROWESS) study, already published in 2001, had shown a significant overall survival benefit at 28 days but had also demonstrated an increased risk of serious bleeding. This latter part was conveniently ignored. Again an error of omission to serve marketing ends.
2. Two other controlled trials, the ADDRESS (Administration of Drotrecogin Alfa [Activated] in Early Stage Sepsis) study and the RESOLVE (Resolution of Organ Failure in Pediatric Patients with Severe Sepsis) study confirmed that increase in risk and resulted in warnings being submitted by Lilly to FDA regarding the use of rhAPC (ibid).
3. Although results of the ADDRESS study were reported in the Oct 2004 ESICM meeting, no mention of the study and its concerns were included in a supplement to the Surviving Sepsis Campaign Guidelines published the following month in *Critical Care Medicine* (ibid). Again a convenient, but glaring error of omission.
4. The efficacy of rhAPC has not been prospectively demonstrated in the patient population for which the drug is currently recommended (ibid) and yet no mention of this was ever given importance to in all the hype over marketing the drug.
5. Despite the persisting scientific controversy surrounding its safety and efficacy, it is included in one of the performance bundles. Implementation of the bundles is being advocated in workshops organized under the auspices of Society of Critical Care Medicine funded by Lilly (ibid). This campaign is directed towards lobbying with state governments in the US to adopt these bundles and efforts to institute these measures internationally are being promoted in a programme convincingly called the ‘Surviving Sepsis Campaign Roadshow’, again also subsidized by Lilly (ibid).
6. While all the marketing campaigns are attractively and aggressively promoted, the concerns of studies which point out risks of serious bleeding and prospective studies which fail to convincingly prove the drug is effective, are conveniently ignored.

Here is the problem; and here is one more example why the pharma image stinks; and where industry has to do a serious rethink to salvage whatever it can of its fast crumbling reputation. As have the rest.

### Quality Of CPGs Varies Considerably

CPGs also have a number of drawbacks that come to attention as we conduct a close study. Let us look at some of them.

Despite the enormous energies invested in guideline authoring, the quality of individual guidelines varies considerably (Shiffman *et al*, 2003). There are eight ‘desirable attributes’ of CPGs according to the Institute of Medicine (IOM): Validity, Reliability, Reproducibility, Clinical Applicability, Clinical Flexibility, Clarity, Documentation, Development by a Multidisciplinary Process and Plans for Review (Field and Lohr, 1992).

Documenting desirable attributes is one thing; following them or offering evidence that they are indeed so, quite another. Moreover, critical information that would attest to validity or would document fulfillment of the other IOM criteria is regularly absent from published guidelines (Shiffman *et al.,* 2003).

The same authors quote the evidence of Shaneyfelt, Mayo-Smith and Rothwangi (1999) who found, in an evaluation reported in the *JAMA* of 279 guidelines developed by U.S. medical specialty societies, that guidelines published in peer-reviewed medical literature do not adhere to established methodological standards. They further quote Grilli, Magrini, Penna, Mura and Liberati's *Lancet* article (2000) which found that, of 431 guidelines produced by specialty societies, 82% did not apply explicit criteria to grade the scientific evidence that supported their recommendations, 87% did not report whether a systematic literature search was performed and 67% did not describe the type of professionals involved in guideline development.

If this were not enough, they further opine that systematic reviews of guidelines for drug therapy have confirmed marked variation in quality: like the one of Graham *et al* (2001) published in the *CMAJ*; or for management of depression by Littlejohn *et al* (1999); or for osteoporosis by Cranney *et al.* (2002). Both nonadherence to methodologic standards and failure to document development activities contribute to this variation (Shiffman *et al.*, 2003).

What are the reasons for this inability to adhere to established methodological standards? Why is critical information that would attest to validity regularly absent from CPGs? Why did 82% not use explicit criteria to grade the scientific evidence that supported their recommendations? Why were 87% not in a position to report whether a systematic literature search was performed? Why did 67% not describe the type of professionals used in guidelines development?

#### Why is there such marked variation in the quality of guidelines?

Of course, the obvious answer is the varied capability of the guidelines developers themselves. Which, hopefully, can be remedied with greater knowledge and stricter adherence to standards. But is it only lack of knowledge that prompts such aberration? One would expect guidelines developers to go out of their way to prove how the process they follow is clear and evidence based. Their reputation is involved. If you, or we, were to lay down a guideline of any nature for peers to follow, we would make it doubly sure that we justify our action by proper evidentials. Why and how, can such a basic need be sidetracked? It can only be because:

1. Those responsible for guideline development do not care for such evidentials. This is not likely, since evidence is the corner stone of their recommendation. They know they cannot get away with that.
2. They do not have the necessary strong evidentials to back their recommendations. In which case the lack of evidence production, lack of explicit criteria, lack of literature search and inability to follow methodological standards can be understood. *Understood,* not *justified*. This is the most likely possibility.
3. They are sure no one is going to ever question them as to its lack, since they occupy strong positions or have established strong credentials. Such bravado can come only if a less than honest motive guides an activity. When self-interest guides, logical thinking, rationality and ethical conduct take a back seat. On occupying strong positions, either in associations or research or even in academia, one believes one can camouflage self-interest by riding roughshod over lack of evidence. Unfortunately or rather, fortunately, the rules of scientific evidence and patient welfare do not allow this game to proceed far.

Which means CPG panelists often do not have strong evidentials to back their recommendations, but do so nevertheless, because what they say remains current coin in the field and they believe they can bulldoze their way into acceptance by the prescribing community, oblivious as it is to their covert motives and impressed as it is by their overt credentials.

How then can CPGs remain an objective consensus of expert opinion on treatment? How can it reduce the possibility of inappropriate care being delivered? How can it become a distillate of biomedical wisdom at a certain point in time? And how can it translate complicated research findings into actionable recommendations of clinical care that are worthy of implementation?

For remedying this situation, we will have to look into what such malafide self-interest can be. What needs are being served by recommending without producing evidence? Why are guidelines developers not revealing conflicts of interest?

The obvious answer is because recommending is necessary, whether there is strong evidence to support it or not. Evidence maybe present, but it is not strong enough and therefore needs to be concealed. But, then, why is recommending necessary at all? The crucial need this serves has to be carefully unraveled. The section that follows should, hopefully, do it for us.

### CPGs Serving Industry Interests

Let us look at a survey (Choudhry, Stelfox and Detsky, 2002) of 192 authors of clinical practice guidelines endorsed by North American and European societies on common adult diseases published between 1991 and July 1999, which considered issues like: nature and extent of interactions of authors with drug manufacturers; disclosure of relationships in published guidelines; prior discussion among authors regarding relationships; beliefs regarding whether authors’ own relationships or those of their colleagues influenced treatment recommendations in guidelines. Their results are worth a close study:

Eighty-seven percent of authors had some form of interaction with the pharmaceutical industry. Fifty-eight percent had received financial support to perform research and 38% had served as employees or consultants for a pharmaceutical company (Choudhry, Stelfox and Detsky, 2002).

What does this mean? Nearly 9 out of 10 authors (87%) of CPG had interactions with industry, 6 out of 10 (58%) received financial support and 4 out of 10 (38%) had been employees or consultants to concerned industry. How can industry interests then not be well taken care of in CPGs?

On average, CPG authors interacted with 10.5 different companies. Overall, an average of 81% (95% confidence interval, 70%-92%) of authors per CPG had interactions (ibid).

Interaction with different companies, because different companies’ drugs were involved. 8 out of 10 authors had such interactions per CPG means the minority of 2 could be easily bypassed when they made recommendations unsuited to connected industry.

Similarly, all of the CPGs for 7 of the 10 diseases included in our study had at least 1 author who had some interaction (ibid).

Which means at least one person who could take industry interests into consideration was present in every CPG.

Fifty-nine percent had relationships with companies whose drugs were considered in the guideline they authored and of these authors, 96% had relationships that predated the guideline creation process (ibid).

6 out of 10 (59%) had relationships with the company whose drug was being considered and these relationships were from much before the CPG was laid down. Which means they were long time associates of related companies and would work to maintain the commercial interests of their concerned sponsors so as to continue the association much longer in the future.

Fifty-five percent of respondents indicated that the guideline process with which they were involved had no formal process for declaring these relationships. In published versions of the CPGs, specific declarations regarding the personal financial interactions of individual authors with the pharmaceutical industry were made in only 2 cases (ibid).

Since the formal need to declare conflict of interest in CPG was not necessary, authors could feel it was all right for them to work for laying down guidelines while subtly and even otherwise, promoting concerned industry interests. In fact, since there was no such need, it suited them admirably.

Seven percent thought that their own relationships with the pharmaceutical industry influenced the recommendations and 19% thought that their coauthors’ recommendations were influenced by their relationships (ibid).

7% thinking their relationship affected their work was denial at its best, for without denial, how would they function? That the denial broke down somewhat was when they judged others a bit less charitably when they attribute motives to 19% of them. A few further studies in this direction would make things clearer, provided the respondents are more honest with their answers.

Any suggestion that financial ties with industry can affect their work is met with an understandable outrage and the proper noises. But the subtle and not so subtle influences of largesse can hardly be denied. And researchers can, with only some difficulty, claim to be a breed apart when it comes to promoting self-interest:

Many researchers profess that they are outraged by the very notion that their financial ties to industry could affect their work. They insist that, as scientists, they can remain objective, no matter what the blandishments. In short, they cannot be bought. What is at issue is not whether researchers can be "bought", in the sense of a quid pro quo. It is that close and remunerative collaboration with a company naturally creates goodwill on the part of researchers and the hope that the largesse will continue. This attitude can subtly influence scientific judgment in ways that may be difficult to discern. Can we really believe that clinical researchers are more immune to self-interest than other people? (Angell, 2000)

All in all, about 60% (59%, to be exact) reported that they had financial ties with the companies whose drugs were considered (Choudhry, Stelfox and Detsky, 2002). This is a finding that casts some serious doubt on the credibility of this important pillar of modern clinical practice.

## Witch Hunt Counterproductive

If 60% reported they had financial interests, what about the rest? Well, we have no way of knowing either way, so they must get the benefit of doubt. But it makes sense to probe whether some disclosers were not made and how many were those.

A word of caution here. This should not become a witch-hunt and our veiled accusations should not offend genuine CPG researchers. That would be counterproductive. For, if such genuine workers get dissuaded, we would do a distinct disservice by this exercise and leave the terrain clear for further malevolence by the black sheep. But we must note that the latter, like Trojan Horses, camouflage intentions and mingle with genuine researchers so well it becomes difficult to distinguish the genuine from the pretender.

Of course the argument can also be forwarded that just because some have commercial interests does not necessarily mean they have compromised their credibility. But how do they prove it is so, for the onus of proof lies with them here? Have they demonstrated, ever, that they wrote or recommended only on merit, even if it compromised the commercial interests of their funding agency? And the recommendation was such as to make a major impact on CPG even at the risk of forgoing benefits to their sponsors? In other words, the recommendation against or neglecting the benefits of, their sponsors were not only cosmetic or trivial? Proof of such nature alone can be convincing and offer some reassurance that things are not that bad after all. That of course does not mean they find fault where there is none. But it is important they expose fault where there is and not look askance as negative results are sidelined or positive results trumpeted. And, in any case, do not act as planted protagonists sabotaging authentic guidelines work by insinuating marketing needs of sponsors that masquerade as evidence based medicine.

A greater discussion of this in the future, and studies highlighting its nuances, would be in perfect order.

## Urgent Reparative Action

So, we realize that because CPGs are accepted as objective consensus of evidence, there is so much more a temptation to use it to serve industry interests. We have to find means by which this temptation avoids playing havoc with scientific integrity, biomedical advance and patient welfare.

We need some urgent reparative action. At least three can be implemented in the light of the foregoing discussion:

### Disclose conflict of interest of CPG authors

One of them is disclosure of financial conflict of interest by authors of CPGs and that too prior to CPG development, with an appropriate ethical review process in place to take suitable action. Of course, one that is not industry funded. Which itself may turn out to be a tall order.

In fact the above study makes similar recommendations in its conclusion:

***Although the response rate for this survey was low, there appears to be considerable interaction between CPG authors and the pharmaceutical industry. Our study highlights the need for appropriate disclosure of financial conflicts of interest for authors of CPGs and a formal process for discussing these conflicts prior to CPG development (Choudhry, Stelfox and Detsky, 2002).***

Although the response rate to their survey was low for obvious reasons, it need not detract from the burden of their conclusions. Moreover, a formal process to discuss conflicts *before* CPGs are developed is a worthy suggestion too, so that they are minimized, if not obliterated altogether. Although, as noted earlier, it is easier said than done. Of course, disclosure on publication will make readers aware of the conflict of interest, nevertheless. But that may amount to closing the stable gate after the horses have bolted.

If a hallowed institution like CPG shows cracks because of this, well, so be it*.* For only then would the malaise be identified and proper reparative action initiated.

### Reject publication with even one conflicted co-author

Another suggestion worth consideration is not to accept for publication any consensus statements (like CPGs, for example), reviews, commentaries etc, in which even one of the authors has a significant financial conflict of interest. This is what the *CMAJ* has to say and it perhaps echoes what many other ethically concerned journals may also feel:

In the face of the growing evidence that financial conflicts of interest bias expert recommendations in favour of sponsors’ products, this Journal (along with most major medical journals) will not accept for publication consensus statements, narrative reviews, commentaries and similar types of articles that recommend drugs, devices, laboratory tests or other interventions for which at least one of the authors has a significant financial conflict of interest (CMAJ, 2005; See also CMAJ, 2004).

### Use of tools like NGC Guideline Summary Sheet

The need to adhere to clear-cut and strict criteria while forming a guideline is another useful step. In this connection, the National Guideline Clearinghouse (NGC) Guideline Summary Sheet is an important primary tool which contains all the key attributes used to summarise each guideline. It's a guideline on guidelines and includes items like a Brief Guideline Summary, the Complete Guideline Summary, the Guideline Comparison and the Guideline Synthesis (National Guideline Clearinghouse, 2007).

The next chapter continues with some further measures to remedy the situation.

## Concluding Remarks

Clinical Practice Guidelines are another example of an excellent idea likely to go to seed due to sponsor manipulation and forces of the market place camouflaging as evidence based medicine. The need to weed out conflicted experts and make the process of therapy selection transparent must go hand in hand with laying down clear-cut criteria for guideline formulation and rejection of conflicted submissions by vigilant journal publication policies and editors.
